# Phantom validation of a novel RSA-based impingement metric to assess component-on-component impingement risk

**DOI:** 10.1177/09544119241238950

**Published:** 2024-03-22

**Authors:** Shahnaz Taleb, Jordan S Broberg, Brent A Lanting, Matthew G Teeter

**Affiliations:** 1 Imaging Group, Robarts Research Institute, Schulich School of Medicine and Dentistry, Western University, London, ON, Canada; 2 Division of Orthopedic Surgery, Department of Surgery, University Hospital, London Health Sciences Center, London, ON, Canada

**Keywords:** Total hip arthoplasty, radiostereometric analysis, X-ray image analysis/reconstruction, implants/prosthetics, impingement

## Abstract

Component-on-component impingement in total hip arthroplasty may lead to post-operative complications including dislocation. Despite numerous clinical studies focusing on reducing this risk, assessment methods remain limited to qualitative radiography, finite element analysis, and cadaver studies. There is a need for more precise measurements of impingement in the research setting. We aimed to validate a novel RSA-based impingement metric to measure component-on-component impingement in vivo. A phantom experiment of a standard metal-on-polyethylene total hip system was performed. RSA examinations were performed as typical for a traditional weight-bearing RSA exam for large joints. The phantom was placed in 10 possible impinged positions and one neutral position. Double exposure radiographs were taken to measure repeatability. The closest distance between the skirt of the head and the inner circumference of the acetabular cup liner was measured to assess impingement risk. Distances between the closest point of the hood to the edge of the cup in 10 impinged positions ranged from 0.05 to 1.03 mm, with the average being 0.67 mm. In the neutral position, the distance measured is 11.02 mm. Excellent repeatability was observed, with a standard deviation of 0.03 mm with an *r* value of 0.09. A validated RSA-based risk metric was established to evaluate in vivo hip impingement. A 1 mm threshold may be proposed to define impingement where distances approaching 1.00 mm are at a greater risk of impingement. This simplified metric holds promise for upcoming clinical studies on component-on-component impingement.

## Introduction

Total hip arthroplasty (THA) is a widely successful orthopedic intervention to treat end-stage hip disease, in which restoration of native joint biomechanics is crucial for implant longevity. Hip impingement is a major cause of poor patient outcomes following THA, leading to joint instability, pain, and recurrent dislocations which can subsequently lead to revision.^1–4^ Impingement is contact between the prosthetic femoral neck and the acetabular cup liner (component-on-component) or contact between the greater trochanter and the pelvis (bone-on-bone). Component impingement may lead to limited range of motion and function post-operatively, pain, dislodgement of the modular liner or loosening of the implant due to increased stress on the liner rim, accelerated metal wear, subluxation, and dislocation.^4–7^ Factors leading to impingement include prosthetic design, surgical technique, component malposition, and patient anatomy. Because of its consequences, many clinical studies are looking at methods to reduce risk of impingement following hip arthroplasty.^5,8–10^ Currently, reducing impingement is being achieved via implant design, improved templating, and intra-operative technologies such as navigation.

However, the assessment of impingement in clinical studies is largely limited to qualitative radiographic assessment of the hip joint, finite element analyses, and cadaver studies.^5,11–13^ Even so, because hip impingement is a dynamic process, its prevalence is difficult to assess using traditional clinical evaluations or plain radiographs. Although dynamic radiographic assessments, such as fluoroscopy, have been used to assess pre-operative femeroacetabular impingement and post-operative contact and separation of the hip, no studies have directly assessed impingement. As such, there are no widely accepted radiographic techniques to validate the occurrence of post-operative component impingement. Qualitative assessment of impingement may be acceptable in clinical practice, but there is a need for more precise measurements in the research setting.

Radiostereometric analysis (RSA) is a minimally invasive dual-plane radiographic technique used to monitor three-dimensional movements of musculoskeletal joints. Its high measurement accuracy, of 0.5 mm for translations and 1.15° for rotations, makes RSA the gold standard for implant micromotion tracking in orthopaedic studies.^14–19^ Today, it is conventionally used to investigate implant component micromotion as well as the biomechanics of novel implants.^14,20–22^ In model-based RSA (MBRSA), dual x-rays are analyzed to calculate an optimal match between the virtual projection of a three-dimensional implant model (a reversed engineered or CAD model) with the contours of the implant on the dual-plane radiographs. Once matched, the three-dimensional pose of each implant component is specified, and the implant position is defined. As such, MBRSA provides a potential avenue to assess impingement risk by investigating the proximity of the femoral stem to the acetabular cup, with closer distances being attributed to greater risk and vice versa. In this study, we aimed to validate a novel impingement risk metric using MBRSA.

## Methods

### Phantom set-up

**Figure 1.**
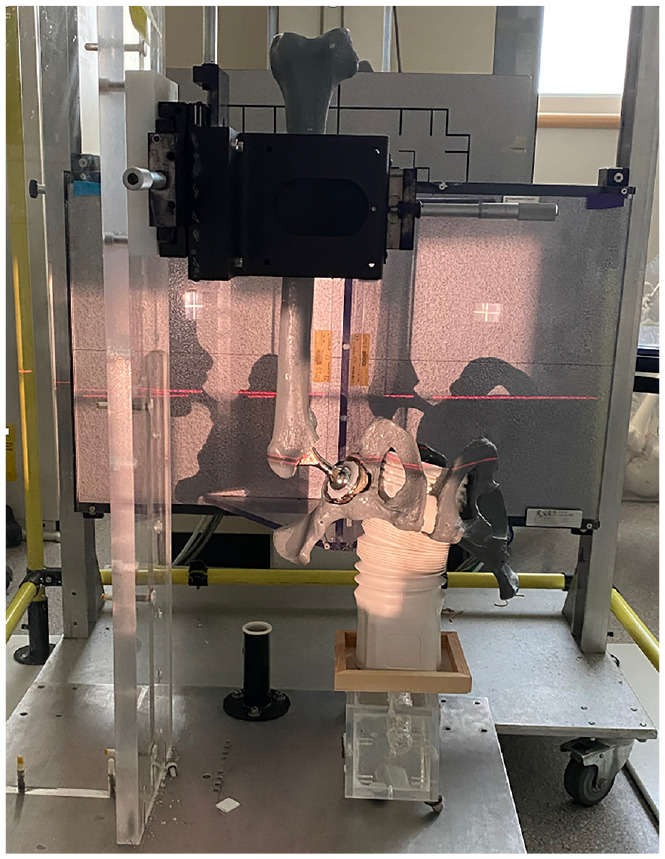
Phantom set-up for radiostereometric analysis imaging.

A Sawbones anatomical model of a male pelvis and femur was created. An experienced surgeon then placed a total hip prosthetic into the model, creating a phantom of a standard THA system implanted in a Sawbone with similar radiographic properties as bone. The hip prosthetic was a standard metal-on-polyethylene total hip system, including a femoral head, femoral stem, 52 mm acetabular cup, and polyethylene liner (Figure 1). The femur was rigidly attached to a composite translation stage (Model M4434, Parker Hannafin, Irwin, USA) and the pelvis was fixed to its base.^17^ The phantom was set-up in such a way that normal anatomy is flipped vertically (pelvis on the bottom, femur on top) such that the Y axis is perpendicular to the ground (Figure 1).

### Radiographic procedure

The radiographic procedure took place in a specialized RSA suite. Within this suite, two X-ray units (Proteus CR/a, GE Medical Systems, Milwaukee, WI, USA) were mounted on the ceiling and positioned at a 20° angle relative to the normal of a uniplanar calibration cage (Cage 43, RSA Biomedical, Umeå, Sweden). The imaging plates used had dimensions of 14 × 17 cm and radiographs were captured using a computed radiography digital system (Capsula XL, FUJIFILM, Tokyo, Japan). The resulting images had a matrix size of 3520 × 4280 pixels, a pixel size of 100 mm, and were represented in 10-bit gray-scale mapping. This setup has been previously employed for phantom validation studies of the hip, knee, and shoulder, and is the standard clinical RSA examination arrangement for large joints.^17,18,23,24^ Joint information and the calibration cage were recorded together in the images. To analyze the captured images, commercial model-based RSA software (version 20190717, RSACore, Leiden, Netherlands) was utilized to determine the 3D projection contour of the implant.

The phantom was placed in 10 possible impingement positions, and one in neutral stance. The impinged positions comprised of four positions of posterior impingement (against the superolateral area of the liner), four of anterior impingement, one in superior impingement, and one in inferior impingement. Double exposure images were taken to measure repeatability of our method, resulting in 22 image pairs. An impingement metric was developed based on the poses extrapolated from the RSA software.

### Impingement metric

**Figure 2.**
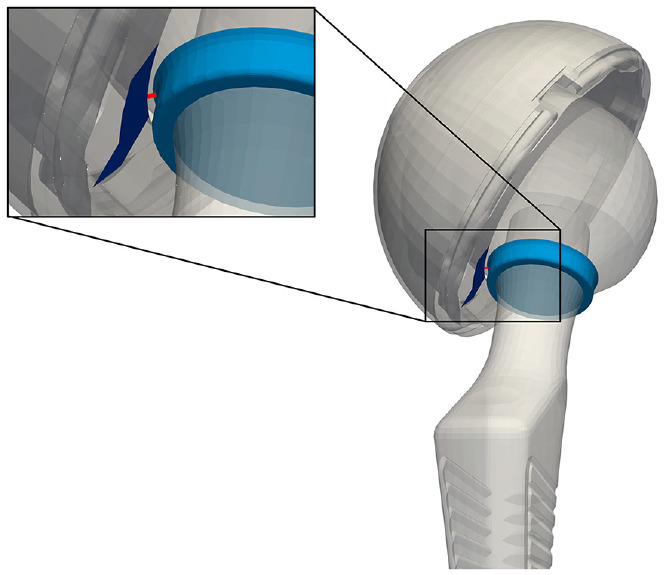
Representation of impingement distance measured as described, as the distance (red) between femoral head skirt (light blue) and the inner circumference of the cup liner (navy).

The impingement metric was calculated using MATLAB. The impingement metric measures the closest distance between the femoral stem—or skirt of the head in cases where skirted heads are used—and the inner circumference of the acetabular cup liner (Figure 2). In essence, since component impingement most often occurs as contact between the polyethylene liner and along the femoral stem at a distance equal to the radius of the acetabular cup, the impingement metric measured the distance between the point on the stem closest to the polyethylene liner and the tangential plane of the liner. Implant component poses from RSA were then used to transform femoral stem, femoral head, and acetabular cup STLs to the imaged positions. The polyethylene liner model was manually placed into the acetabular component. The magnitude of the vector between the closest points on the femoral stem (or head skirt) and the inner circumference of the polyethylene liner represents the impingement distance.

### Statistical analysis

Repeatability of MBRSA was calculated based on methods described by Langlois and Hamadouche, and the 2013 ASTM guidelines.^25,26^ Measurements of precision were given by repeatability standard deviation, s_r_, and repeatability limit, *r*, of image exposures. All statistical analyses were conducted using Prism version 7.0 (GraphPad Software Inc., La Jolla, USA).

## Results

For the 10 imaged impingement risk positions, distances between the closest point of the skirt to the edge of the cup ranged from 0.05 to 1.03 mm, with the average being 0.67 mm. In the neutral position, the distance measured is 11.02 mm. the impingement risk assessment tool yielded suitable repeatability results, with a standard deviation of 0.03 mm and a repeatability limit of 0.09, comparable to the accuracy of RSA, which has been reported to range from 0.047 mm to 0.121 mm in in vitro studies.^27^ Means and exposure measurements of all imaged positioned are described in Table 1.

**Table 1.** Means and repeatability measurements between RSA exposures of the impingement metric in 10 different impingement positions.

Positions	First exposure (mm)	Second exposure (mm)	Difference (mm)	Mean (mm)
1	0.70	0.67	0.02	0.69
2	0.60	0.58	0.02	0.59
3	0.03	0.08	0.05	0.05
4	0.30	0.30	0.00	0.03
5	1.05	1.02	0.03	1.03
6	1.04	1.05	0.01	1.05
7	0.93	0.91	0.02	0.92
8	0.91	0.88	0.03	0.90
9	0.37	0.34	0.03	0.35
10	0.85	0.91	0.06	0.88
11*	10.99	11.04	0.05	11.02

* Non-impinged position, at neutral.

## Discussion

Component impingement is a problematic occurrence that needs to be more properly understood. Despite the growing interest in component impingement following total hip arthroplasty, there are no widely accepted radiographic techniques to quantifiably measure the occurrence of impingement. In this work, we validated a novel impingement tool using MBRSA that allows for the assessment of component impingement in vivo.

The proposed tool in this study allows us to quantifiably assess impingement risk following total hip arthroplasty in vivo, which, to our knowledge, is the first of its kind. Component impingement studies often rely on qualitative assessment of patient radiographs.^11^ Previous studies have also used surrogate measurements for impingement risk, such as sagittal implant measurements of ante-inclination and sacral acetabular angle.^12^ Other methods of measuring impingement include range-of-motion testing in clinic visits or at the time of implant revision.^13^ As such, to the best of our knowledge, there is currently no validated way of measuring component impingement in vivo. Our proposed metric of impingement risk may therefore be valuable in post-operative impingement studies. Since the average impingement metric across all 10 exams was 0.67 mm with the greatest distance being 1.03 mm, a threshold of 1.00 mm is proposed to define impingement, with risk of impingement increasing as distances approach the threshold.

MBRSA presents very small biases and excellent repeatability for all translation planes. Repeatability of our metric is consistent with previous model-based rotation studies.^19^ It is however important to note the potential of errors that may be carried over into the impingement measurements. First, there is the slight dimensional variation between the implant’s CAD model and the implant itself from casting and manual polishing, which makes achieving complete alignment between the detected and true contour of the implant unattainable. Moreover, the polyethylene liner present in standard metal-on-polyethylene implants acetabular components is not detected in the radiographic images and were accordingly removed from contour detection but reintroduced in impingement measurements. The polyethylene model was manually placed snugly into the acetabular component as would be implanted, allowing room for potential error.^28^ Fitting the liner into the acetabular cup was done as an iterative process where model opacities are reduced and fitted in such a way that the liner sits flush with the acetabular cup. In cases with elevated liners, modeling will be done in the same way, where the liner and cup models are fit optimally at their base. Although errors may be introduced, errors attributing to a fraction of a millimeter or degree is marginally relevant in the assessment of component impingement risk given our proposed 1-mm threshold. Lastly, a limitation of our current study is the use of our hip phantom with no soft-tissue coverage, creating an ideal scenario for RSA analysis. The inclusion of soft-tissue in RSA phantom experiments have reported to more closely mimic in vivo accuracy results. However, previous studies have reported that errors introduced due to soft-tissue coverage are negligible, with a standard deviation of about 0.06 mm.^29–32^

## Conclusions

We successfully created a tool to assess component impingement in vivo using MBRSA, a significant step forward in the understanding component impingement in patients following total hip arthroplasty.
